# Autophagy Genes for Wet Age-Related Macular Degeneration in a Finnish Case-Control Study

**DOI:** 10.3390/genes11111318

**Published:** 2020-11-06

**Authors:** Jussi J. Paterno, Ali Koskela, Juha M.T. Hyttinen, Elina Vattulainen, Ewelina Synowiec, Raimo Tuuminen, Cezary Watala, Janusz Blasiak, Kai Kaarniranta

**Affiliations:** 1 Department of Ophthalmology, University of Eastern Finland, 70211 Kuopio, Finland; jussi.paterno@uef.fi (J.J.P.); ali.koskela@uef.fi (A.K.); juha.hyttinen@uef.fi (J.M.T.H.); elivat@student.uef.fi (E.V.); 2 Department of Ophthalmology, Kuopio University Hospital, 70029 Kuopio, Finland; 3 Faculty of Biology and Environmental Protection, Department of Molecular Genetics, University of Lodz, 90-136 Lodz, Poland; ewelina.synowiec@biol.uni.lodz.pl (E.S.); janusz.blasiak@biol.uni.lodz.pl (J.B.); 4 Helsinki Retina Research Group, University of Helsinki, 00014 Helsinki, Finland and Department of Ophthalmology, Kymenlaakso Central Hospital, 48100 Kotka, Finland; raimo.tuuminen@helsinki.fi; 5 Department of Haemostatic Disorders, Chair of Biomedical Sciences, Medical University, 92-215 Lodz, Poland; cezary.watala@umed.lodz.pl

**Keywords:** aging, autophagy, degeneration, macula, neovascularization

## Abstract

Age-related macular degeneration is an eye disease that is the main cause of legal blindness in the elderly in developed countries. Despite this, its pathogenesis is not completely known, and many genetic, epigenetic, environmental and lifestyle factors may be involved. Vision loss in age-related macular degeneration (AMD) is usually consequence of the occurrence of its wet (neovascular) form that is targeted in the clinic by anti-VEGF (vascular endothelial growth factor) treatment. The wet form of AMD is associated with the accumulation of cellular waste in the retinal pigment epithelium, which is removed by autophagy and the proteosomal degradation system. In the present work, we searched for the association between genotypes and alleles of single nucleotide polymorphisms (SNPs) of autophagy-related genes and wet AMD occurrence in a cohort of Finnish patients undergoing anti-VEGF therapy and controls. Additionally, the correlation between treatment efficacy and genotypes was investigated. Overall, 225 wet AMD patients and 161 controls were enrolled in this study. Ten SNPs (rs2295080, rs11121704, rs1057079, rs1064261, rs573775, rs11246867, rs3088051, rs10902469, rs73105013, rs10277) in the *mTOR* (Mechanistic Target of Rapamycin), *ATG5* (Autophagy Related 5), *ULK1* (Unc-51-Like Autophagy Activating Kinase 1), *MAP1LC3A* (Microtubule Associated Protein 1 Light Chain 3 α), *SQSTM1* (Sequestosome 1) were analyzed with RT-PCR-based genotyping. The genotype/alleles rs2295080-G, rs11121704-C, rs1057079-C and rs73105013-T associated with an increased, whereas rs2295080-TT, rs2295080-T, rs11121704-TT, rs1057079-TT, rs1057079-T, rs573775-AA and rs73105013-C with a decreased occurrence of wet AMD. In addition, the rs2295080-GG, rs2295080-GT, rs1057079-TT, rs11246867-AG, rs3088051-CC and rs10277-CC genotypes were a positively correlated cumulative number of anti-VEGF injections in 2 years. Therefore, variability in autophagy genes may have an impact on the risk of wet AMD occurrence and the efficacy of anti-VEGF treatment.

## 1. Introduction

Age-related macular degeneration (AMD) is the most common cause of permanent sharp and color vision loss in the elderly which has a significant socioeconomic burden on patients and their caregivers and societies [1]. The AMD number is expected to increase by at least 15% in the coming decades [2]. The prevalence of AMD in Caucasian populations is reported as high as one third or more at the age of 80 and more years [1,2,3]. In addition to genetic and epigenetic components [4,5,6,7], many cardiovascular and lifestyle risk factors are associated with AMD development [8].

Most commonly, AMD starts in its dry form, which may progress to wet (exudative) AMD in 10–20% of cases [1]. The clinical hallmark of wet AMD is the pathogenic choroidal neovascularization (CNV) that may leak fluids into the retina. Exudative AMD is a rapidly progressive sight-threatening condition and should be treated aggressively with intravitreal anti-vascular epithelial growth factor (anti-VEGF) injections [9,10]. Despite this often-effective treatment, 10% of wet AMD patients present a low visual outcome within two-year follow-up [11,12]. The varying anti-VEGF treatment responses have been explained by genetic factors, the timing of the initiation of treatment, and the choice of drug used [9,13,14,15].

The cellular pathogenesis of AMD includes impaired proteolytic clearance in the macular retinal pigment epithelium (RPE). In the normal visual cycle, RPE cells are constantly exposed to light and phagocytose the lipid-rich outer photoreceptor segments. This combined with high metabolic activity results in an enhanced oxidative stress [16]. Chronic oxidative stress makes proteins vulnerable to damage and leads to the detrimental accumulation of aberrant proteins. Clinical signs for the protein aggregation are the accumulation of lysosomal lipofuscin in RPE and extracellular drusen deposits between RPE and choriocapillaris. Currently, emerging evidence suggests that cellular proteostasis is disturbed in AMD and autophagy, as its crucial mechanism, plays an important role in the various stages of AMD development and progression [16,17,18,19,20,21].

Autophagy is a lysosomal clearance mechanism to degrade damaged, aged or unneeded proteins in cells. Detailed autophagy signaling has been reviewed elsewhere [22]. Briefly, the mechanistic target of rapamycin (mTOR), autophagy related genes (ATGs), the serine/threonine uncoordinated-51-like kinase 1 (ULK1), microtubule-associated protein light chain 3 (LC3) and sequestosome-1 (p62/SQSTM1) are key molecules to regulate selective autophagy. It is initiated by the formation of a double-membrane vesicle, autophagosome, enclosing material to be degraded (cargo) that is delivered to the lysosome, where degradation and recycling occur [23]. mTOR controls the initiation signals of autophagy. The active ULK1 participates in autophagosome initiation to create the phagophore. ATG7 activates ATG5, which forms a complex with ATG12 and ATG16L1 leading to the extension of the phagophoric membrane in autophagic vesicles. This complex is necessary for LC3-I conjugation to PE (phosphatidylethanolamine) to form LC3-II conjugate. LC3 has ubiquitin and p62/SQSTM1 binding sites that connect autophagy to the proteasomal clearance system. Once specific cargos are ubiquitinated and recognized by LC3 and p62/SQSTM1, they undergo autolysosomal degradation. Autophagy impairment, caused by the depletion of the core autophagy genes *ATG5* and *ATG7*, was associated with an AMD-like phenotype in mouse RPE cells [18]. This phenotype was manifested by RPE thickening, hypertrophy or hypotrophy, pigmentary abnormalities and the accumulation of oxidized proteins. Oxidative stress is a canonical stimulus to induce autophagy in RPE cells [19,20,21,24,25]. Studies on RPE cells from AMD donors and mice with AMD-like phenotype suggest autophagy involvement in AMD pathogenesis [20]. However, the autophagosome formation in late AMD was reported to occur at a lower rate than in early stages of this disease. This study also revealed that chronic oxidative stress decreased autophagic flux. Autophagy can prevent retinal cells from the damaging effects of oxidative stress [19,20,21,26].

Since most of the autophagy studies for AMD pathology have been done in cell culture and animal models, we wanted to unravel how the single nucleotide polymorphisms (SNPs) of autophagy genes have been associated with wet AMD and the outcomes of anti-VEGF treatments in a Finnish case-control cohort.

## 2. Materials and Methods

### 2.1. Study Population and Treatment

**Figure 1:**
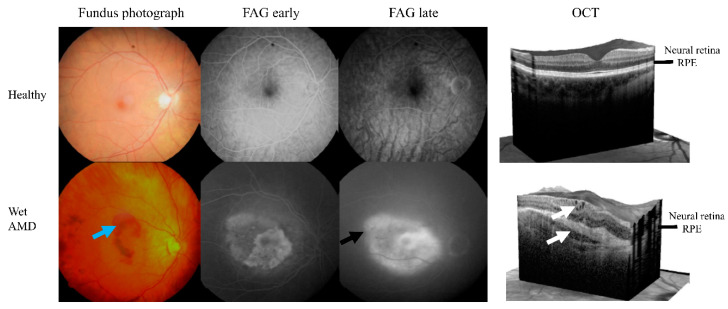
Color fundus photographs, fluorecein angiography (FAG) and optical coherent tomography (OCT) images from healthy and wet AMD cases. Images retrieved from participants enrolled in this study, wet AMD patient had a TT allele of rs73105013. The clinical hallmarks of wet AMD are hemorrhages (blue arrow), fluorescein leaking in a late FAG (black arrow) and intra/subretinal fluids in OCT (white arrows). RPE means retinal pigment epithelium layer.

The study population consisted of 225 wet AMD patients and 161 controls (Table 1). The mean age of the patients was 78.4 years (range 74.5–84.5), whereas the mean age of controls was 74.1 years (68.6–78.7). The patient group contained 70 males and 155 females; these numbers in the control group were 52 males and 109 females. The controls were individuals without AMD or any other retinal disease, who were undergoing cataract operation and were selected as described earlier [27]. The criteria for patient selection were based on CNV in optical coherence tomography (OCT) and/or fluorescein angiography (FAG) (Figure 1). No patient reported any genetic disease and diabetes mellitus was an exclusion criterion. All AMD patients were subjected to an examination in the Department of Ophthalmology of Kuopio University Hospital, involving best-corrected Snellen equivalents visual acuity (VA; baseline changed between 0.05 and 1.0), biomicroscopy, fundus photographs (Canon CX-1 Hybrid Retinal Camera, Canon, Tokyo, Japan), FAG (Canon CX-1) and/or OCT (SPECTRALIS OCT2, Heidelberg Engineering, Heidelberg, Germany) (Figure 1). Real world data (RWD) were monitored for up to two years. Finnish national guidelines for modified *pro re nata* (PRN) were applied in the follow-up and treatments of wet AMD patients [28].

Clinical data included blood pressure, anti-cholesterol, anti-coagulant and anti-platelet drugs. Body mass index (BMI) and smoking history were also examined (Table 1).

Ethics Committee of the Kuopio University Hospital (42/2014) has approved the study and the tenets of the Declaration of Helsinki were followed. All participants signed an informed consent form.

Some 10 mL aliquots of whole blood were taken into a 3 mL tube containing EDTA, shaken gently 10 times, and kept at room temperature for 30–60 min prior to 3200× *g* centrifugation at 20 °C for 15 min. DNA was extracted from the peripheral blood leucocytes with QiAamp DNA Blood Midi kit (Qiagen, Valencia, CA, USA) according to the manufacturer’s instructions. The quality and the amount of the extracted genomic DNA were measured with NanoDrop Microvolume spectrophotometer model ND-1000 (ThermoFisher Scientific, Wilmington, MA, USA).

The public domain of the single-nucleotide polymorphism database at the National Center for Biotechnology Information and the related literature to identify potentially functional polymorphisms in autophagy genes were used for SNP selections. The selected polymorphisms have known distributions in various European populations. SNP selection favored those with a minor allele frequency no less than 3%. Moreover, we selected polymorphisms at different positions in the relevant genes. There were polymorphisms located directly in the core promoter and thousands base pair upstream from the transition start site. We selected both exonic and intronic locations as well as the 3′ untranslated region. This diversity in the locations of SNPs within genes ensures their potential various functionality. Genotyping was performed using TaqMan SNP Genotyping Assays (Thermo Fisher Scientific, Foster City, CA, USA) (Table 2) according to the manufacturer’s instructions. Briefly, genotyping was carried out in a total volume of 15 µL containing 1× TaqMan Genotyping Master Mix (Thermo Fisher Scientific), 1× SNP Genotyping Assay mix and 2 ng of genomic DNA in a 96-well plate format. PCR reactions were carried out with 2720 Thermal Cycler (Thermo Fisher Scientific) and the fluorescein labels were read using QuantStudio 5 (Thermo Fisher Scientific).

### 2.2. Statistical Analysis

The descriptive data are shown as the mean ± SD or as the number of patients in each category. The normality of the studied group was verified with the Shapiro–Wilk test, the homogeneity of variance was checked with Brown–Forsythe test. Accordingly, either the unpaired Student’s *t* test or Mann–Whitney *U* test was used. In some bivariate and multivariate analyses, we used the approach of resampling with replacement (the bootstrap-boosted versions of the tests, 10,000 iterations) to make sure that the revealed differences were not detected by a pure chance.

An unconditional multiple logistic regression model was used to calculate the associations between the studied polymorphisms and the occurrence of a disease (with or without stratification for sex). The results are shown as odds ratios (ORs) with a 95% confidence interval (±95% CI). In addition, the significant outcomes were further validated with the use of two approaches: the bootstrap-boosted multiple logistic regression (resampling with replacement, 10,000 iterations) and the cross-validated logistic regression that corresponds to the *d*-jackknife technique with the patient group as the modelled class. This was intended to overcome any possible bias related to relatively low sample sizes. The goodness-of-fit of logistic regression models showing a significant discrimination between controls and patients was estimated with the Hosmer–Lemeshow test. The analysis of collected data was done in Statistica 12 (Statsoft, Tulsa, OK, USA), SigmaPlot 11.0 (Systat Software Inc., San Jose, CA, USA), Resampling Stats Add-in for Excel v.4 (Arlington, VA, USA) and StudSize3.02 (CreoStat HB, Västra Frölunda, Sweden; used for power analysis).

## 3. Results

Autophagy-related genes (*mTOR*, *ATG5*, *ULK1*, *MAP1LC3A*, and *SQSTM1*) SNPs cover the genes and 10 kbp flanking regions both up- and downstream (Table 2). Based on their linkage disequilibrium (LD) structure, the seven SNPs cover 90% of the allelic variation within these regions, while three SNPs showed disequilibrium (Table 3). The total genotyping rate in the individuals was >90%. Differences in call rates for each genotype between cases and controls were tested and no significant (*p* < 0.05) differences were detected in genes that showed agreement with Hardy–Weinberg equilibrium (HWE) (Table 3). However, rs2295080, rs11121704 and rs573775 deviations were detected that significantly (*p* < 0.05) departed from the HWE disequilibrium.

**Figure 2:**
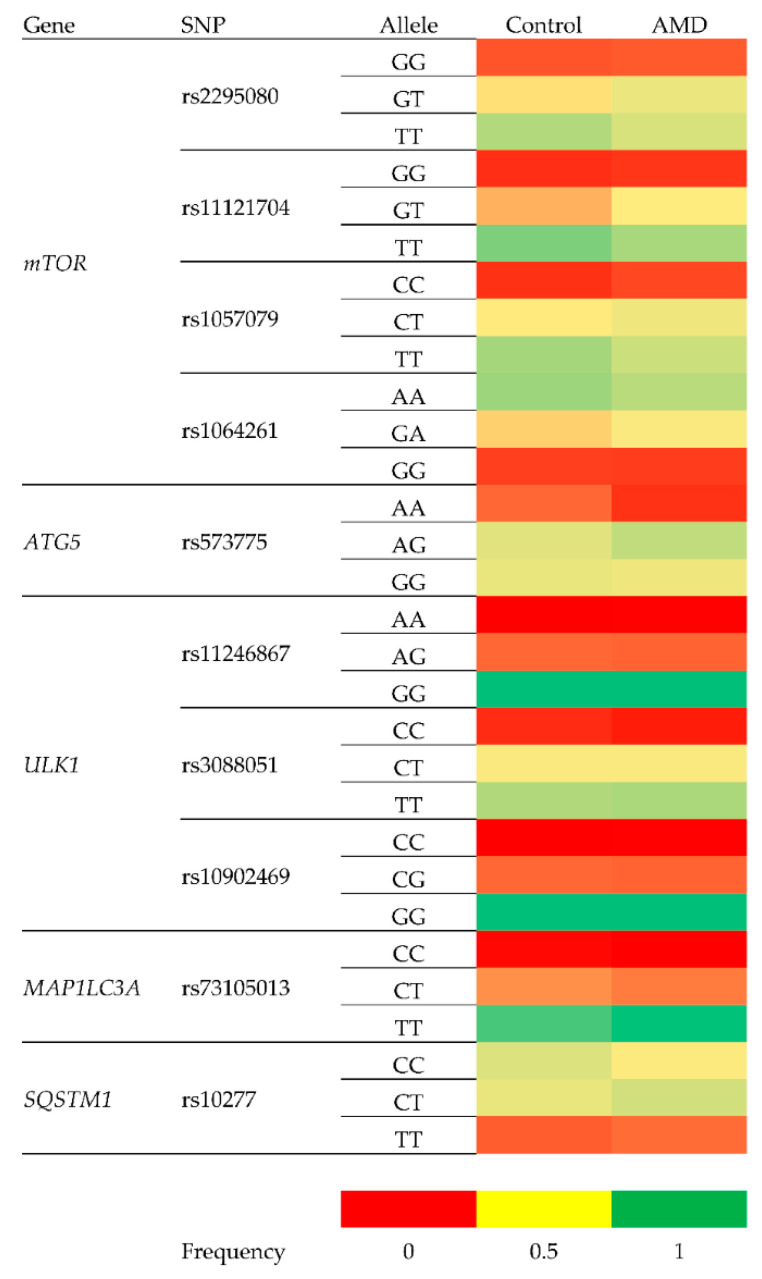
A heatmap indicating the differences in allele frequencies of SNPs analyzed between healthy controls and AMD patients.

As shown in Table 4, the following genotypes/alleles were associated with an increased risk of wet AMD: rs2295080-G (adjusted), rs11121704-C (adjusted), rs1057079-C (crude and adjusted) and rs73105013-T (crude and adjusted). The protective associations were observed for rs2295080-TT (crude and adjusted), rs2295080-T, rs11121704-TT (adjusted), rs1057079-TT (crude and adjusted), rs1057079-T (crude and adjusted), rs573775-AA (crude) and rs73105013-C (crude and adjusted). In general, these associations showed ORs of about 1.5 in cases of positive associations and not less than 0.4 in their negative counterparts. A heatmap was generated for the visualization of different allele frequencies between healthy controls and wet AMD patients (Figure 2).

Since the amount of anti-VEGF intravitreal injections varies between patients and with personalized demands, we were curious to analyze the connections of autophagy gene SNPs and the amount of cumulative injections during two years of treatment. Our results showed that the rs2295080-GG, rs2295080-GT, rs1057079-TT, rs11246867-AG, rs3088051-CC and rs10277-CC genotypes were associated with two years of a cumulative number of injections (0.13 < R < 0.14 Spearman’s correlation coefficient) (Table 5).

## 4. Discussion

AMD is a multifactorial, multi-compartmental complex eye disease. The importance of genetic factors and their variability were confirmed in many studies (reviewed in [6]). However, the penetrance of many gene variants is very low, and one of the challenges of complex disease is to capture the extent of processes that may be perturbed. Genome-wide association studies (GWAS) have shown that mutations in the complement system genes are strongly associated with the risk of AMD. However, those SNPs do not differentiate AMD phenotypes, progression, or treatment responses. Phenotypic, demographic, environmental, genetic, and molecular risk factors have been used to predict disease progression, but personalized risk may differ from that determined by commonly accepted risk factors [29]. Therefore, rare variants should be considered in the prediction of AMD progression and treatment responses. Moreover, it is assumed that the risk genes are expressed in the retinal tissue and are locally involved in AMD mechanisms [30].

Since AMD is strongly associated with disturbed proteostasis [16,23], we targeted our gene analysis to autophagy genes that are not strong hits in GWAS analyses [6]. To our knowledge, we showed for the first time that autophagy gene polymorphisms were moderately associated with wet AMD development and anti-VEGF treatment responses. The T alleles of the *mTOR* gene loci rs2295080, rs11121704 and rs1057079 indicated OR 0.6 protection against wet AMD. The ATG5 rs573775 A allele and the LC3 rs731105013 C allele SNPs showed a similar trend in the prevention of wet AMD. These SNP associations revealed that autophagy cascaded from the autophagy initiation to the flux termination and may have a regulatory role in the development of wet AMD. The rs73105013 T allele might be a useful genotyping marker in deeper wet AMD risk analysis. Interestingly, the *mTOR* rs2295080 G allele associated with both an increased wet AMD and weaker anti-VEGF treatment response once the cumulative amount of injections for two years was calculated. According to the Finnish wet AMD guidelines, a *pro re nata* protocol was used for anti-VEGF injections [28]. To show the complexity of the genetic association studies, the *mTOR* rs1057079 T allele had a protective sign to develop wet AMD, but was associated with a weaker anti-VEGF treatment response, as it was related to the number of given injections. Similar observations were made for the protective genotype of rs800292 variant of the complement H (CFH) gene with a poor anatomical response [31]. The p.69A > S polymorphism for the homozygous T risk allele required more anti-VEGF injections over the 48-month follow-up [14]. A cumulative effect of the high-risk alleles in *CFH*, *ARMS2*, and *VEGFA* was associated with a younger age of AMD onset in combination with a poor response rate to anti-VEGF treatment [32]. A limited contribution of common genetic variants to variability in wet AMD treatment response was observed, while rare protein-altering variants in the *C10orf88* and *UNC93B1* genes associated with a worse response to anti-VEGF therapy [15]. Rare variants, including SNPs in autophagy genes, may play an important role in personalized anti-VEGF treatment responses.

Numerous AMD-related risk factors have been documented, but the evidence and strength of association is variable depending on ethnicity and study design. Our Finnish cohort shows that smoking history and the use of anti-cholesterol drugs are associated with wet AMD. One systemic review and meta-analysis showed moderate and consistent risk associations with higher BMI, history of cardiovascular disease, and hypertension [33]. A recent French study indicated a high risk for the incidence of early AMD in individuals with high plasma high-density lipoprotein (HDL) cholesterol levels and confirmed the high risk for progression from early to advanced AMD in heavy smokers and carriers of the *CFH* Y402H at-risk genotypes [34]. In numerous studies, smoking is reported as a modifiable risk factor in AMD. It is associated with a 2–4-fold increased risk for any form of AMD [29]. Our results are in line with those which previously reported the role of smoking in AMD development. The use of anti-cholesterol drugs was also clearly associated with wet AMD in this study. The age-related eye disease study (AREDS) report reveals that statin use is not significantly associated with the progression to late AMD in the AREDS2 participants [35]. In our material, anti-cholesterol drugs were not classified and plasma HDL levels were not analyzed. Interestingly, autophagy has a key role in the clearance of cholesterol droplets and high cholesterol levels may decrease autophagy [36,37]. In addition, autophagy, glucose, lipid, and energy metabolism regulator adiponectin receptor 1 rs10753929 *ADIPOR1* variant was associated with advanced AMD in the Finnish population [27].

An increased BMI is found to be associated with the increased chance of developing AMD in some studies, while some other studies show no association [29]. We did not observe association between increased BMI and wet AMD. Although the possible involvement of hypertension in AMD has been well documented in the literature, it is not always found to be a risk factor for AMD and anti-hypertensive medication is not proven to have a positive effect on AMD [29]. We analyzed only blood pressure and drug use without finding any association with wet AMD prevalence. A limitation of our study is that we did not analyze BMI development, hypertension disease history or its severity level and we did not classify anti-hypertensive drugs. In recent years, anti-coagulation and anti-platelet drugs have been considered to exacerbate wet AMD [38]. We found no association between the use of low-dose anti-coagulating or anti-platelet drug use and wet AMD, which is in line with the recent reports [39,40,41].

The number of injections, regimens and patient compliance changes between patients. We used Finnish national guidelines for modified PRN which allows a different anti-VEGF drugs and injection regimen. By identifying the prognostic biomarkers of AMD that could be used to select the individual drug treatment and follow-up protocol, the quality and safety of treatment and patient compliance could be improved. This would also more precisely allocate available resources, especially as the need for AMD patients care increases as the population ages. Variability in autophagy-related genes in the Finnish population may moderately contribute to wet AMD pathogenesis and therapy. The Finnish population has reduced diversity and homogeneity, which is beneficiary for gene mapping studies. Since we used RT-PCR-based SNP genotyping, further work should include the whole-genome sequencing of the samples with subsequent analysis and public deposition of the acquired datasets. We believed that autophagy is one important target to develop pharmacogenetics and personalized medicine for AMD.

## 5. Conclusions

Autophagy genes are associated with wet AMD and the efficacy of anti-VEGF treatment in a Finnish Case-Control Study.

## Figures and Tables

**Table 1:** Some clinical and lifestyle features of wet age-related macular degeneration patients and controls enrolled in this study.

Feature	Control (*n* = 161)	Wet AMD (*n* = 225)	*p* ^3^
Age (years)	74.1 ± 6.3	78.4 ± 6.9	**0.000**
Sex (male/female)	52/109	70/155	0.368
BMI (mean)	25.7 ± 4.5 ^1^	26.2 ± 4.1 ^2^	0.161
Smoking			**0.001**
Non-smoker	93 (57.8%)	140 (62.2%)	
Occasionally	16 (9.9%)	26 (11.6%)	
Smoker	6 (3.7%)	27 (12.0%)	
No information	46 (28.6%)	32 (14.2%)	
Medication			
Blood pressure	115 (79.3%) ^1^	164 (76.3%) ^2^	0.340
Anti-cholesterol	77 (53.1%) ^1^	93 (43.5%) ^2^	**0.011**
Anticoagulant	43 (29.5%) ^1^	64 (30.2%) ^2^	0.832
Antiplatelet	66 (44.6%) ^1^	109 (50.9%) ^2^	0.094

^1^*n* = 115 (BMI, body mass index), 145 (blood pressure), 145 (anti-cholesterol), 146 (anticoagulant), and 148 (antiplatelet); ^2^ *n* = 186 (BMI), 215 (blood pressure), 214 (anti-cholesterol), 212 (anticoagulant), and 214 (antiplatelet) ^3^ values of *p* < 0.05 are in bold mean ± SD for age and BMI.

**Table 2:** Single-nucleotide polymorphisms of autophagy-related genes in this study.

Polymorphism	Gene	Change	Location within Gene
rs2295080	*mTOR—Mechanistic Target of Rapamycin Kinase*	g.11262571G > T, C	5′UTR (promoter)
rs11121704		g.11233902C > A, T	Intron
rs1057079		c.1437C > T	Exon
rs1064261		c.2997C > T	Exon
rs573775	*ATG5—Autophagy Related 5*	g. 106316991G > A	Intron
rs11246867	*ULK1—Unc-51 Like Autophagy Activating Kinase 1*	g. 131893472G > A	2 KB Upstream Variant
rs3088051	*ULK1—Unc-51 Like Autophagy Activating Kinase 1*	g. 131922463T > C	3′UTR
rs10902469		g. 131893588G > A, C	2 KB Upstream Variant
rs73105013	*MAP1LC3A—Microtubule Associated Protein 1 Light Chain 3 α*	g. 179837731T > A, C	Intron
rs10277	*SQSTM1—Sequestosome 1*	g. 179837731T > A, C	3′UTR

**Table 3:** Distributions of the genotypes of polymorphisms of autophagy-related genes in wet AMD patients and controls tested for agreement with Hardy–Weinberg equilibrium.

Polymorphism	Control		AMD	
	Chi-square	*p* ^1^	Chi-square	*p* ^1^
rs2295080	**6.720**	**0.035**	1.201	0.548
rs11121704	**6.090**	**0.048**	2.192	0.334
rs1057079	1.464	0.481	0.673	0.714
rs1064261	5.081	0.079	0.898	0.638
rs573775	0.456	0.796	**6.555**	**0.038**
rs11246867	1.044	0.593	0.109	0.947
rs3088051	0.220	0.896	0.401	0.818
rs73105013	1.884	0.390	1.987	0.370
rs10277	0.668	0.716	0.033	0.984
rs10902469	1.044	0.593	0.109	0.947

^1^ The values of *p* in bold correspond to polymorphisms whose distributions depart from Hardy–Weinberg equilibrium (HWE).

**Table 4:** Genotype and allele distribution of the polymorphisms of autophagy-related genes in wet AMD patients and controls.

Genotype/Allele	Frequency		Crude OR (95% CI)	*p*	Adjusted OR ^1^ (95% CI)	*p*
	Control	AMD				
**rs2295080 148/216 (Control/AMD cases)**						
GG	0.12	0.12	1.07 (0.57–2.00)	0.836	1.28 (0.65–2.53)	0.474
GT	0.32	0.42	1.51 (0.98–2.34)	0.062	1.49 (0.94–2.38)	
TT	0.56	0.46	**0.661 (0.435–1.004)** **0.664 (0.454–0.971) ^2^**	**0.052** **0.035**	**0.621 (0.398–0.968)** **^2^ 0.617 (0.393–0.968)^0.651^**	**0.035** **0.036**
G	0.22	0.27	1.296 (0.941–1.785)	0.113		
T	0.28	0.23	0.772 (0.560–1.063)	0.113	**0.709 (0.503–0.998)** **^2^ 0.703 (0.493–0.995)^0.725^**	**0.048** **0.049**
**rs11121704 152/209 (Control/AMD cases)**						
CC	0.07	0.09	1.15 (0.53–2.52)	0.720	1.23 (0.54–2.82)	0.616
CT	0.26	0.33	1.489 (0.940–2.357) 1.481 (0.936–2.345) ^2^	0.089 0.093 ^2^	1.609 (0.985–2.628) ^2^ 1.634 (0.982–2.717)^0.459^	0.056 0.059
TT	0.67	0.58	0.671 (0.436–1.034) 0.663 (0.433–1.016) ^2^	0.071 0.059 ^2^	**0.613 (0.386–0.972)** **^2^ 0.605 (0.375–0.975)^0.717^**	**0.037** **0.039**
C	0.16	0.21	1.34 (0.94–1.91)	0.102	1.447 (0.993–2.109) **^2^ 1.467 (1.016–2.119)^0.479^**	0.054 **0.041**
T	0.33	0.29	0.74 (0.52–1.06)	0.102	0.691 (0.474–1.007) **^2^ 0.681 (0.474–0.985)^0.809^**	0.054 **0.041**
**rs1057079 161/217 (Control/AMD cases)**						
CC	0.07	0.10	1.47 (0.71–3.04)	0.293	1.69 (0.77–3.71)	0.191
CT	0.33	0.41	1.37 (0.90–2.10)	0.141	1.39 (0.88–2.18)	0.153
TT	0.60	0.49	**0.653 (0.434–0.984)** **0.654 (0.435–0.982) ^2^**	**0.041** **0.041 ^2^**	**0.621 (0.402–0.961)** **^2^ 0.620 (0.396–0.972)^0.675^**	**0.032** **0.037**
C	0.20	0.25	**1.413 (1.021–1.955)** **1.403 (1.017–1.936) ^2^**	**0.037** **0.039 ^2^**	**1.500 (1.059–2.123)** **^2^ 1.507 (1.059–2.144)^0.612^**	**0.022** **0.023**
T	0.30	0.25	**0.708 (0.511–0.979)** **0.707 (0.513–976) ^2^**	**0.037** **0.035 ^2^**	**0.667 (0.471–0.944)** **^2^ 0.661 (0.470–0.929)^0.869^**	**0.022** **0.017**
**rs1064261 138/215 (Control/AMD cases)**						
GG	0.09	0.09	0.94 (0.45–1.96)	0.874	0.98 (0.45–2.13)	0.961
GA	0.30	0.37	1.42 (0.90–2.24)	0.131	1.50 (0.92–2.44)	0.101
AA	0.61	0.53	0.74 (0.48–1.14)	0.174	0.70 (0.44–1.10)	0.123
G	0.19	0.23	1.19 (0.85–1.68)	0.313	1.25 (0.87–1.80)	0.229
A	0.30	0.27	0.84 (0.59–1.18)	0.313	0.80 (0.55–1.15)	0.230
**rs573775 160/214 (Control/AMD cases)**						
AA	0.14	0.07	**0.492 (0.253–0.954)** **0.487 (0.246–0.964) ^2^**	**0.036** **0.039 ^2^**	0.611 (0.304–1.227)	0.165
AG	0.44	0.53	1.42 (0.94–1.14)	0.092 ^2^	1.31 (0.85–2.03)	0.221
GG	0.42	0.40	0.92 (0.61–1.39)	0.699	0.92 (0.59–1.43)	0.717
A	0.29	0.30	0.90 (0.67–1.21)	0.493	0.94 (0.68–1.29)	0.707
G	0.21	0.20	1.11 (0.82–1.50)	0.493	1.06 (0.77–1.46)	0.707
**rs11246867 161/217 (Control/AMD cases)**						
AA	0.00	0.00	2.1 × 10^7^ (0–0)	0.997	1.6077 × 10^10^ (0–0)	1.000
AG	0.15	0.15	0.98 (0.55–1.72)	0.933	0.95 (0.52–1.73)	0.860
GG	0.85	0.85	0.99 (0.56–1.40)	0.970	1.00 (0.55–1.83)	0.994
A	0.07	0.08	1.04 (0.61–1.79)	0.881	1.05 (0.59–1.85)	0.878
G	0.42	0.42	0.96 (0.56–1.65)	0.881	0.96 (0.54–1.69)	0.878
**rs3088051 149/216 (Control/AMD cases)**						
CC	0.07	0.05	0.71 (0.30–1.73)	0.455	0.69 (0.26–1.84)	0.462
CT	0.36	0.38	1.10 (0.72–0.69)	0.665	1.13 (0.71–1.80)	0.599
TT	0.58	0.57	0.98 (0.65–1.50)	0.941	0.96 (0.61–1.50)	0.855
C	0.21	0.22	0.96 (0.68–1.35)	0.822	0.98 (0.68–1.41)	0.903
T	0.29	0.29	1.04 (0.74–1.46)	0.822	1.02 (0.71–1.48)	0.903
**rs73105013 156/210 (Control/AMD cases)**						
CT	0.21	0.18	0.79 (0.47–1.32)	0.366	0.73 (0.42–1.23)	0.252
TT	0.76	0.82	1.53 (0.92–2.53)	0.102	1.65 (0.96–2.83)	0.069
C	0.12	0.09	**0.597 (0.376–0.949)** **0.601 (0.376–0.960) ^2^**	**0.029** **0.033 ^2^**	**0.561 (0.344–0.919)** **^2^ 0.565 (0.337–0.947)^0.695^**	**0.021** **0.030**
T	0.38	0.41	**1.674 (1.054–2.660)** **1.686 (1.052–2.703) ^2^**	**0.029** **0.030 ^2^**	**1.779 (1.089–2.910)** **^2^ 1.776 (1.078–2.927)^0.993^**	**0.021** **0.024**
**rs10277 136/212 (Control/AMD cases)**						
CC	0.46	0.35	0.657 (0.426–1.015) 0.656 (0.429–1.004) ^2^	0.059 0.052 ^2^	0.658 (0.414–1.047)	0.077
CT	0.41	0.49	1.34 (0.87–2.06)	0.182	1.28 (0.80–2.03)	0.297
TT	0.13	0.16	1.27 (0.68–2.38)	0.450	1.41 (0.72–2.76)	0.316
C	0.44	0.42	0.75 (0.54–1.03)	0.071	0.74 (0.53–1.03)	0.074
T	0.06	0.08	1.34 (0.97–1.36)	0.078	1.36 (0.97–1.91)	0.074
**rs10902469 161/217 (Control/AMD cases)**						
CG	0.15	0.15	1.34 (0.97–1.86)	0.934	0.95 (0.52–1.73)	0.086
GG	0.85	0.85	0.99 (0.56–1.74)	0.970	1.00 (0.55–1.83)	0.994
C	0.08	0.08	1.04 (0.61–1.79)	0.881	1.05 (0.59–1.85)	0.888
G	0.42	0.42	0.96 (0.56–1.65)	0.881	0.95 (0.54–1.69)	0.880

^1^ Odds ratio (OR) adjusted for sex and the usage of antiplatelet and cholesterol-lowering drugs; for significant comparisons the ^2^ means the bootstrap-boosted OR (resampling with replacement, 10,000 iterations); all OR values without bootstrap analysis were calculated using the cross-validation algorithm. Statistical power; (1−β) (calculated at α = 0.05) for significant comparisons given in superscripts. *p* < 0.05 along with the corresponding ORs are in bold.

**Table 5:** Multiple correlation between the cumulative number of injections (median of six injections) for two years in wet AMD patients and genotypes of polymorphisms in autophagy-related genes.

Polymorphism	Genotype	R	*p*
rs2295080	**GG**	**0.137**	**0.040 ^1^**
	**GT**	**0.136**	**0.041 ^1^**
	TT	0.088	0.377
rs11121704	CC	0.089	0.362
	CT	0.099	0.313
	TT	0.089	0.367
rs1057079	CC	0.100	0.309
	CT	0.106	0.281
	**TT**	**0.131**	**0.049 ^1^**
rs1064261	GG	0.093	0.344
	GA	0.129	0.053
	AA	0.111	0.257
rs573775	AA	0.083	0.398
	AG	0.105	0.287
	GG	0.107	0.274
rs11246867	AA	0.106	0.114
	**AG**	**0.133**	**0.045 ^1^**
	GG	0.106	0.278
rs3088051	**CC**	**0.148**	**0.026 ^1^**
	CT	0.104	0.290
	TT	0.083	0.398
rs73105013	CC		
	CT	0.105	0.281
	TT	0.105	0.281
rs10277	**CC**	**0.131**	**0.049 ^1^**
	CT	0.086	0.380
	TT	0.087	0.194
rs10902469	CC		
	CG	0.084	0.391
	GG	0.084	0.391

^1^ Bootstrapped. Adjusted to sex, age and the use of anticholesterolemic therapy. Statistically significant values are shown in bold. Cases impossible to calculate are shown by a blank row.
